# Increased utilisation and quality: a focus on inequality and a learning health system approach—explaining Nepal’s success in reducing maternal and newborn mortality 2000–2020

**DOI:** 10.1136/bmjgh-2023-011836

**Published:** 2024-05-02

**Authors:** Sudha Sharma, Oona Maeve Renee Campbell, William Edward Oswald, Dadhi Adhikari, Punya Paudel, Bibek Lal, Loveday Penn-Kekana

**Affiliations:** 1 Private Consultant, Kathmandu, Nepal; 2 Infectious Disease Epidemiology and International Health, London School of Hygiene and Tropical Medicine, London, UK; 3 Department of Disease Control, London School of Hygiene & Tropical Medicine, London, UK; 4 Global Health Division, RTI International, Research Triangle Park, North Carolina, USA; 5 South Asian Institute for Policy Analysis and Leadership, Kathmandu, Nepal; 6 Government of Nepal, Kathmandu, Nepal; 7 Family Welfare Division, Government of Nepal Ministry of Health and Population, Kathmandu, Nepal

**Keywords:** Maternal health, Health systems evaluation, Health policies and all other topics, Health policy

## Abstract

**Introduction:**

Maternal mortality in Nepal dropped from 553 to 186 per 100 000 live births during 2000–2017 (66% decline). Neonatal mortality dropped from 40 to 21 per 1000 live births during 2000–2018 (48% decline). Stillbirths dropped from 28 to 18 per 1000 births during 2000–2019 (34% decline). Nepal outperformed other countries in these mortality improvements when adjusted for economic growth, making Nepal a ‘success’. Our study describes mechanisms which contributed to these achievements.

**Methods:**

A mixed-method case study was used to identify drivers of mortality decline. Methods used included a literature review, key-informant interviews, focus-group discussions, secondary analysis of datasets, and validation workshops.

**Results:**

Despite geographical challenges and periods of political instability, Nepal massively increased the percentage of women delivering in health facilities with skilled birth attendance between 2000 and 2019. Although challenges remain, there was also evidence in improved quality and equity-of-access to antenatal care and childbirth services. The study found policymaking and implementation processes were adaptive, evidence-informed, made use of data and research, and involved participants inside and outside government. There was a consistent focus on reducing inequalities.

**Conclusion:**

Policies and programmes Nepal implemented between 2000 and 2020 to improve maternal and newborn health outcomes were not unique. In this paper, we argue that Nepal was able to move rapidly from stage 2 to stage 3 in the mortality transition framework not because of what they did, but how they did it. Despite its achievements, Nepal still faces many challenges in ensuring equal access to quality-care for all women and newborns.

**WHAT IS ALREADY KNOWN ON THIS TOPIC**

**WHAT THIS STUDY ADDS**

- The study adds to the existing body of evidence by synthesising qualitative and quantitative data on change over a 20-year period, with a focus on positive achievements. We worked closely with past and current Ministry of Health and Population officials to gain better insights into the policy processes associated with Nepal’s achievements and sought to test mid-level theories for these successes.

**HOW THIS STUDY MIGHT AFFECT RESEARCH, POLICY OR PRACTICE**

- Many countries have good policies, but their effective implementation remains elusive and planned results are not achieved. Evidence emerging from Nepal can guide countries to effectively implement and scale-up successful programmes. It may also help Nepal fine-tune its policies and programmes for greater impetus in achieving future health goals.

## Introduction

 According to global estimates, maternal mortality in Nepal dropped from 553 to 186 per 100 000 live births during 2000–2017 (66% decline), neonatal mortality from 40 to 21 per 1000 live births during 2000–2018 (48% decline) and stillbirths from 28 to 18 per 1000 births during 2000–2015 (34% decline).^1^ This resulted in Nepal moving from phase 2 to phase 3 in the maternal–neonatal–stillbirth transition model.^2^ Nepal outpaced global and South Asian regional declines in maternal and newborn mortality and stillbirths, after considering its gross national income (GNI) per capita. This success was achieved despite Nepal’s unique geography, 80% of the population still living in rural areas in 2021,^3^ periods of political instability, and a devastating earthquake in 2015 which seriously impacted health and other infrastructure.

As a result of these achievements, Nepal was identified as one of seven positive outlier countries to be included in the Maternal and Newborn Health (MNH) Exemplars project that is based on the premise that useful lessons can be learnt from countries which have achieved notable declines in maternal and neonatal mortality.^4^ Nepal was also identified as a positive-outlier country for its achievements in Child Survival^5^ and reduction of Stunting.^6^ This paper describes the key mechanisms that emerged, using a mixed methods case study approach, as explaining Nepal’s success in reducing maternal and neonatal mortality. This study was carried out alongside similar studies in India, Bangladesh, Ethiopia, Senegal, Niger, Morocco and six states in India.

The article draws on the Learning Health Systems literature—that argues that successful health systems show a commitment to learning, and ‘connection between information, deliberation and action’. And that moreover health systems are strengthened and perform better if they focus on ‘developing institutional and human capacities’ at different levels within the health system, and creating space for ‘reflective practice and learning within routine processes’.^7 8^

### Context

At the start of our time period of interest in 1990, Nepal had a multiparty democracy with a constitutional monarchy. A Maoist insurgency began in 1996, and a 10-year civil war ensued. During the conflict, neither side targeted health facilities, with some exceptions. In 2002, the monarchy dissolved parliament claiming absolute power. A peace-accord in 2006 led to an interim constitution and a Constituent Assembly election, which declared Nepal as a federal democratic republic, abolished the monarchy and elected the country’s first President. The interim period lasted until the new constitution was promulgated (2015) and elections held (2017). The constitution created 1 federal, 7 provincial and 753 local governments, and enshrined health as a human right.

Nepal is landlocked with three ecological zones, the Terai (flat), the hills, and the mountains. Every year, massive monsoon floods and landslides further exacerbate geographic challenges. The country is predominantly rural, with an overall population density (2019) of 203 people per kilometre^2^; the Terai and urban areas are densely populated while the mountains are sparsely populated. The country is culturally diverse with 126 ethnic/caste groups; its population increased from 18.9 million (1990) to 29.1 million (2021), a growth rate of 0.93%. Most people are employed in agriculture (69%), services (19%) and industry (12%).^3^

Nepal has experienced a period of economic growth and was reclassified from a low to a lower middle income country in 2019.^3^ Infrastructure expanded, including roads, transport, communication and access to electricity and water, which can be logically expected to make it easier for women and newborns to reach healthcare

## Methods

Researchers from the South Asian Institute for Policy Analysis and Leadership, including an independent Nepali Principal Investigator (PI) who had been Secretary in the Ministry of Health and Population (MoHP), worked together with researchers from the London School of Hygiene and Tropical Medicine (LSHTM). Current and previous staff of MoHP were involved throughout. Ethical clearance was received from Nepal Health Research Council and LSHTM’s ethics review board.

This was a mixed-method iterative case study. Several approaches and conceptual frameworks were used to guide the work. Within the wider MNH Exemplars project, a holistic conceptual framework was developed to enable researchers from a range of disciplines to consider a broad range of interrelated factors that could lead to improved MNH and survival was developed.^9^ This framework categorised factors into distal (policy, macrolevel context, community-level context), intermediate (programme and service levers and household and individual context) and proximate factors (coverage of effective interventions). This approach was used as a scaffold—although this paper predominantly focuses on intermediate and distal levels of policy, programme and service levers.

We also drew on approaches used in the realist approach—where the focus is not just on the context and the results—but trying to develop mid-level theory/suggested mechanisms to explain outcomes. These theories are then tested and adjusted using available evidence.^10 11^

Specific methods used included a review of grey and published literature; initiation and final validation meetings with key stakeholders to identify themes that contributed to Nepal’s success; a policy-review process; key-informant interviews, focus-group discussions and secondary-data analyses of Demographic and Health Surveys (DHS), Multiple-Indicator Cluster Surveys (MICS) and health management information systems (HMIS) data. Our approach to qualitative research was guided by the Critical Appraisal Skills Programme (CASP) checklist for qualitative research.^12^

As proposed within the realist method, we initially used qualitative data collection to identify potential mechanisms and mid-level theories for how Nepal’s mortality reductions were achieved. We then looked for quantitative evidence, literature or other data to confirm or refute that these were credible mechanisms for the mortality reduction observed. Quantitative analyses of existing data sets were also used to derive temporal trends in mortality, service coverage and utilisation using Stata V.17 and R. Equity analyses were done using ANCq, (a novel content-qualified ANC coverage indicator created and validated using national surveys and based on contact with the health services and content of care received)^13^ and other coveage variables

Qualitative data collection started with the project initiation meeting where we invited key informants identified by the research team or suggested by the Department of Family Welfare to make initial suggestions about mechanisms that had led to Nepal’s success. We then followed up with 25 semistructured interviews, using an interview guide, with past/current government and non-government stakeholders. The initial list of interviewees was developed by the research team, and then a snowball sampling approach was used. Ten focus-group discussions with managers, providers, mothers’ groups and female community health volunteers (FCHVs) (in two purposively selected districts in two provinces in Nepal) were also carried out. A focus group guide was developed exploring explanations for Nepal’s success in reducing maternal and neonatal mortality in the last 20 years.

A useful information source was the annual reports produced by the Department of Health Services, which contained commissioned research results and data analysis as well as reflections and insights from officials working in the health services and programmes. These reports provided insights into the challenges with policy implementation and gave explanations for policy adaptions or changes. Nepal also had several key documents that reflect on the past, take on board global evidence and policy positions, and outline short-term, medium-term and long-term strategies considering the Nepali context; we used these for contemporaneous insights.

At the end of the project, preliminary findings and evidence were presented for validation to a group of 27 stakeholders and reviewed by independent commentators identified by the Family Welfare Division. Three senior MHN experts also critiqued a draft report. Further adjustments to the analysis were made after this process, and insights of other experts were incorporated.

### Reflexivity

The NepaliPI, having previously held a senior position in the government, brought her own insights. She also had close trusting relationships with many of those involved in MHN services. This was immensely useful to the project in terms of access to senior officials and information about processes that happened but that may not have been documented in reports or the literature. This project was looking at explanation of success and we believe this also meant that people were less defensive or reserved about discussing processes and policy when this was the focus.

We were, however, concerned that there may be issues that people may want to raise with an outsider or feel uncomfortable raising to a former colleague. To ensure that this was not unduly biasing the information given—some interviews were also conducted by the UK PI.

This project faced the key methodological challenge of knowing what was casual in terms of Nepal’s achievements in mortality reduction. We, however, developed and tested a range of mechanisms and those presented were ones that were judged the most credible by the research team and by those we presented and discussed our results with.

The qualitative interviews often asked policymakers, managers, staff and women to recall events that happened over a 20-year period. Many of those currently in key positions admitted they had no insight into what had happened 20 years previously or why. Some retired staff were interviewed to attempt to mitigate this. Recall bias was also a challenge. There was also the challenge that many officials were involved in particular aspects of policy—but did not necessarily have a full picture of what happened.

We relied on national-level quantitative data that had been collected using consistent methods over time. This resulted in reliance on a limited range of data from population-based DHS and MICS. Nepal only had one Service Provision Assessment (SPA) (facility survey) in the time period we were studying. While we made some use of the national HMIS, there were limitations in what was measured and how—and we did not have access to the complete data set for the entire period. Political and administrative boundaries changed over time; therefore data were not collected in the same way by the population-based household surveys, making trends in subnational or geographical indicators difficult to assess. Classifications of urban and rural populations in these surveys also changed temporally. While recent (and some past) policy and planning documents are widely available online—this was not the case for those from 20 years ago, and there were insufficient resources to carry out a detailed historical study.

A literature review of papers published between 2000 and 2020 was carried out. Almost all the articles looked at interventions or evaluated policies covered over a limited timescale, usually 2 or 3 years—and, therefore, it was hard to know whether the impact of projects continued beyond the period of the analysis or whether they contributed to achievements over the 20 years.

## Results and discussion

The following mechanisms emerged as key to explaining Nepal’s success in reducing maternal and newborn mortality in the years 2000–2020.

### Mechanism 1: dramatic increase in the percentage of women delivering in health facilities, alongside simultaneous improvement in quality of care within facilities

#### Increased utilisation

‘In the past, most deliveries took place in people’s homes, usually in the cowshed (because labour and delivery were seen as impure). In later years, the deliveries shifted to birthing centres. Now people want better quality of care and come to larger hospitals’. *Retired government official.*

**Figure 1:**
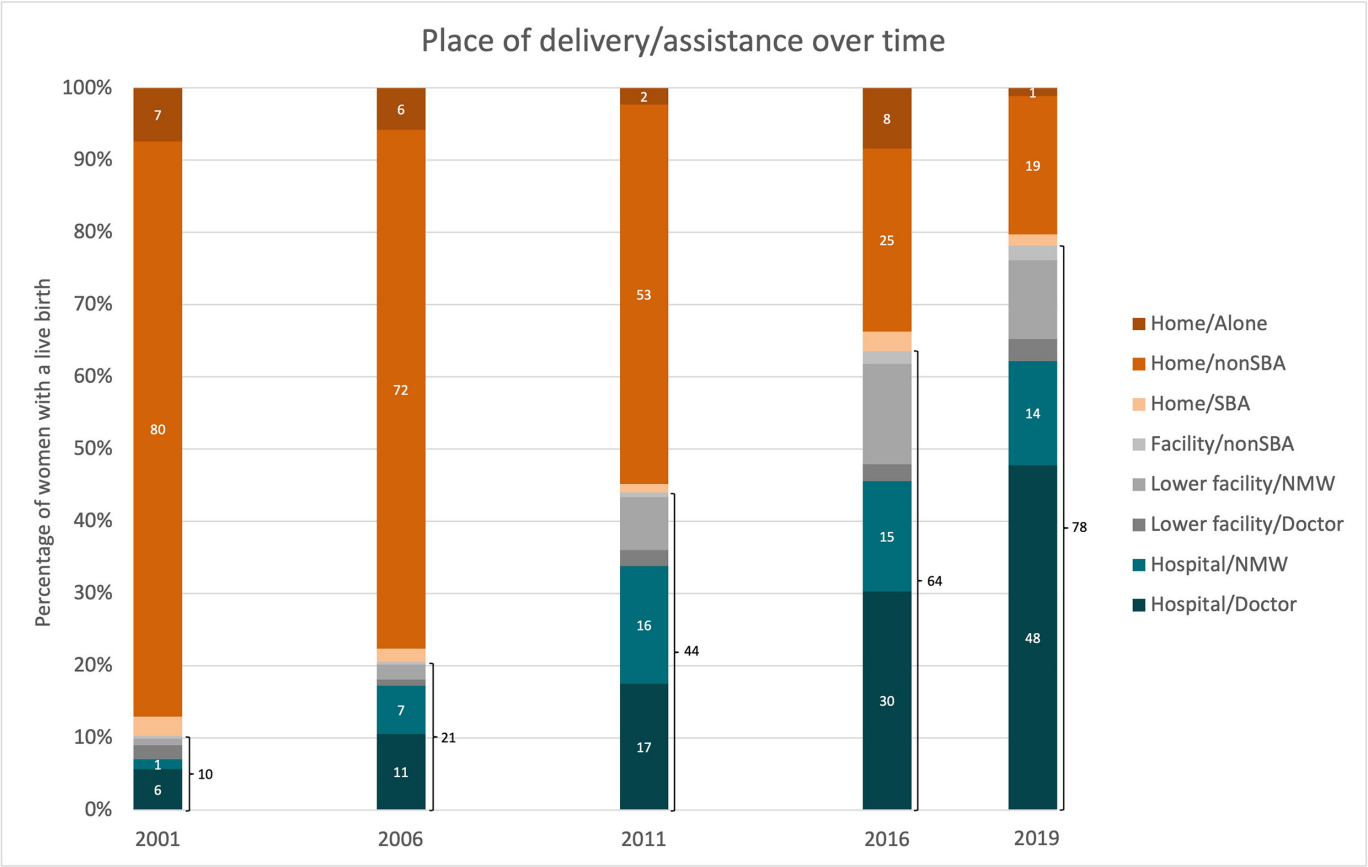
Place of delivery/assistance over time using Demographic and Health Surveys (DHS) & Multiple-Indicator Cluster Surveys (MICS); SBA, skilled birth attendant; NMW, nurse midwife

A clear theme to emerge in both national and district interviews, and from the quantitative analysis, was the dramatic increase in the numbers of women delivering in health facilities, and the cultural and attitudinal changes that led to this. Figure 1 clearly shows the rapid changes in use-patterns of delivery services that happened between 2001 and 2019. In 2001, only 10% of women delivered in a facility, whereas in 2019, it was 78%. Most of the increase was in the public sector.

Figure 1 also confirms the perspective that emerged from the qualitative work that many more women were delivering in higher level facilities with doctors or nurses/midwives assisting. The shift to more skilled birth attendant (SBA) and hospital deliveries can be used as proxies for improved quality-of-care, and the data show more hospital and doctor-attended births. Health workforce data also suggest a concomitant increase in the health workforce, with the ratio of doctors per 10 000 population rising from 2.12 in 2004 to 8.52 in 2021, now exceeding WHO recommended norms.^14 15^ Data were not available on the distribution of these doctors. In focus groups with women, community health workers, and health workers, all confirmed that there has been a huge shift in cultural norms around delivering in facilities.

Several policy processes were identified as having contributed to this achievement—both supply and demand measures were undertaken by government starting in the Safe Motherhood Plan of Action (1994–1997) and followed by the Safe Motherhood Long-Term Plan (2002–2017). It was clear that the government’s policy focus changed over time and differed by geographical region. For example, FCHVs originally played a major role in motivating women to deliver in facilities, but in later years, when communication and societal norms had shifted, they became more involved in community-based newborn care.^16^ The SBA programme started with in-service training but moved to improved preservice training for nurses, and ultimately to formal midwifery certificate and diploma courses. Task shifting for SBA trained doctors and nurses was a major feature of service expansion. Incentives for women to use services started by covering transport costs to facilities, to free healthcare for mothers and babies, and ultimately to cash incentives for attending ANC and Delivery Services. As the national policies changed and developed over time, policies were also adapted in different geographical areas—for example, in remote and rural areas, nurses were trained in anaesthesia, and in some locations, FCHVs were allowed to distribute misoprostol to prevent postpartum haemorrhage.^17 18^

A continuing challenge mentioned by many interviewees was the tension between access and quality—particularly related to maintaining staff and staff skill sets in remote rural facilities where very low numbers of women deliver, and where consequently there are few emergencies.^15^ Key informant interviews, policy documents and HMIS data show that although Nepal initially focused on access, concerns about ensuring quality for both mothers and newborns led local-level governments to increasingly try to ensure women delivered either in hospitals or in birthing centres strategically placed in areas which a large number of women can access and be referred for complications, if needed. The strategy to pay for transport costs, with greater subsidies in remote areas, went alongside a range of emergency transport initiatives, including investment in ambulances, and in exceptional cases using helicopters to transport women in hard-to-reach mountainous area. Maternity waiting homes did not play a significant role in Nepal, with a few exceptions, but there have been calls to review if they have a role.^15 19^ While DHS/MICS data show an increase in women accessing facilities in all social economic groups and all regions—access to services does still remain an issue for women in remote rural areas and among some cultural groups in the flat areas and continues to be a stated priority of the government.

#### Improvements in quality of care

Everyone interviewed, all the policy documents, and much of the published literature identified a need to do more to improve the quality of care being provided, but there is also evidence of achievements in improving the quality of care over the last two decades. Definitions of quality of care, and consistent ways of measuring quality of care over time was a challenge in this project, but we inferred quality by looking at trends in the levels of facilities where women obtained care, the cadres providing care, the timeliness of care (assessed via ANC in the first trimester) and the content of ANC and delivery care (assessed via the proportions of women and newborns receiving interventions of known efficacy).

Figure 1 illustrates that not only did more women delivering in facilities, but that increasingly these facilities are hospitals, which can be assumed to be an indication that the quality of care is improving, as the Nepal SPA shows hospital to have greater readiness and capability than lower level facilities.

**Figure 2:**
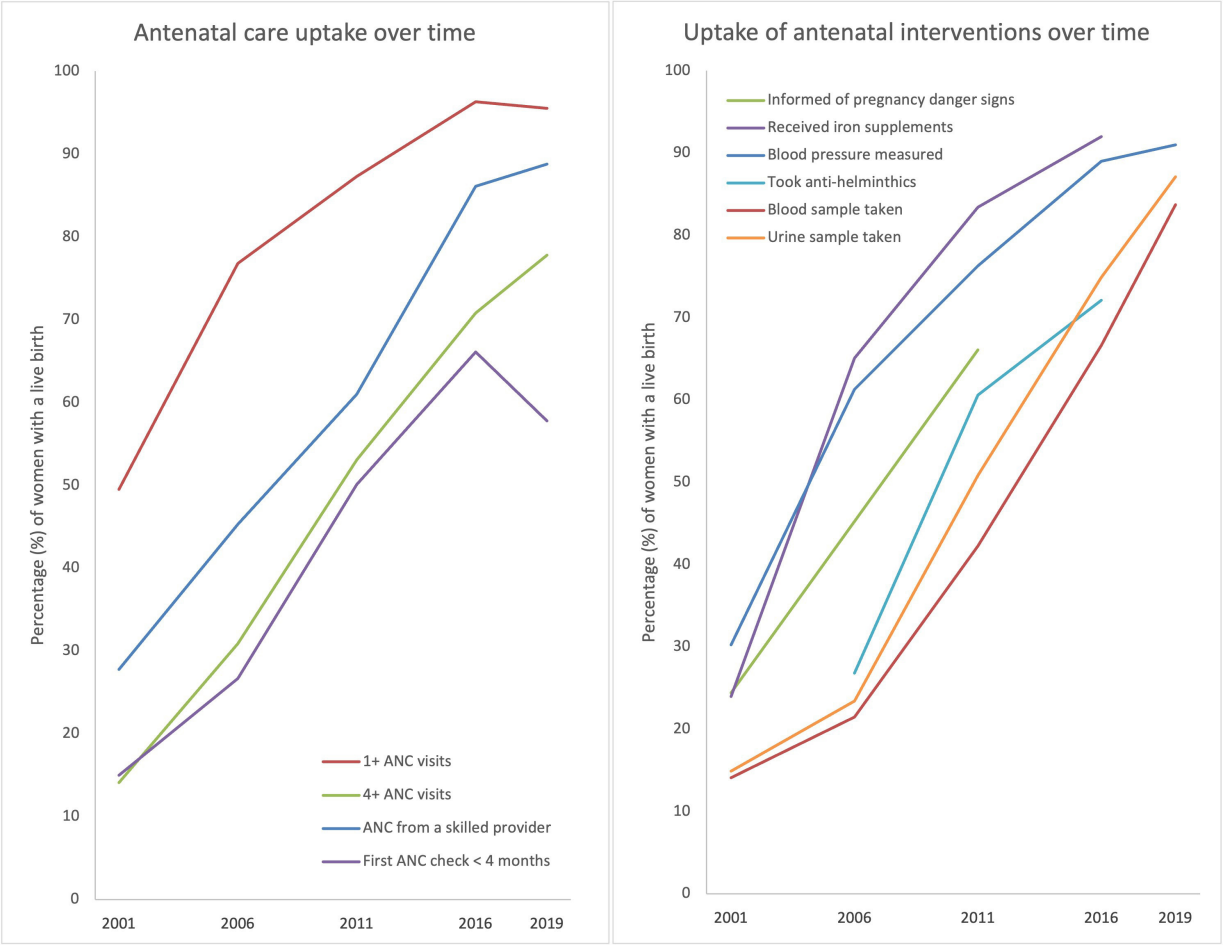
Uptake of antenatal care (ANC) and antenatal interventions in Nepal (2001–2019), from DHS and MICS. Percentages for antenatal interventions (except for provision of iron supplements and intestinal parasite drugs with were measured among all pregnant women) were derived by multiplying the percentages of women with at least one ANC visit by the percentage who received the intervention of interest among women who attended ANC services. DHS, Demographic and Health Surveys; MCIS, Multiple-Indicator Cluster Surveys.

Figure 2 uses DHS and MICS analysis to illustrate that uptake of ANC also increased, including the percentage of women with more than one ANC visits, more than four visits, ANC provided by a skilled provider, and receipt of a first ANC check within the first 4 months. It also shows that based on women’s recall of ANC interventions, there was an increase in women being informed of pregnancy danger signs, receiving iron supplements, having blood pressure taken, taking antihelminthic medications, and having blood and urine samples taken. These results, taken alongside data in figure 1, indicate that an improvement in the quality of care was likely.

### Mechanism 2: consistent commitment in policy and implementation to address a range of inequalities, including the overlapping challenges of geographic access and socioeconomic inequality

A key goal in all government policy documents, and expressed in the Nepal constitution, is the achievement of health-for-all and of reductions in inequality of access. The need to address inequalities, and what had been done to try and achieve this, was a key component of all policy discussions, and a challenge raised in almost all interviews. Coverage, quality and equity are inter-related themes—as coverage approaches universal levels, inequity decreases, even in remote areas. However, apparent equity, for example, via high levels of contact with services may mask inequities in quality.

**Figure 3:**
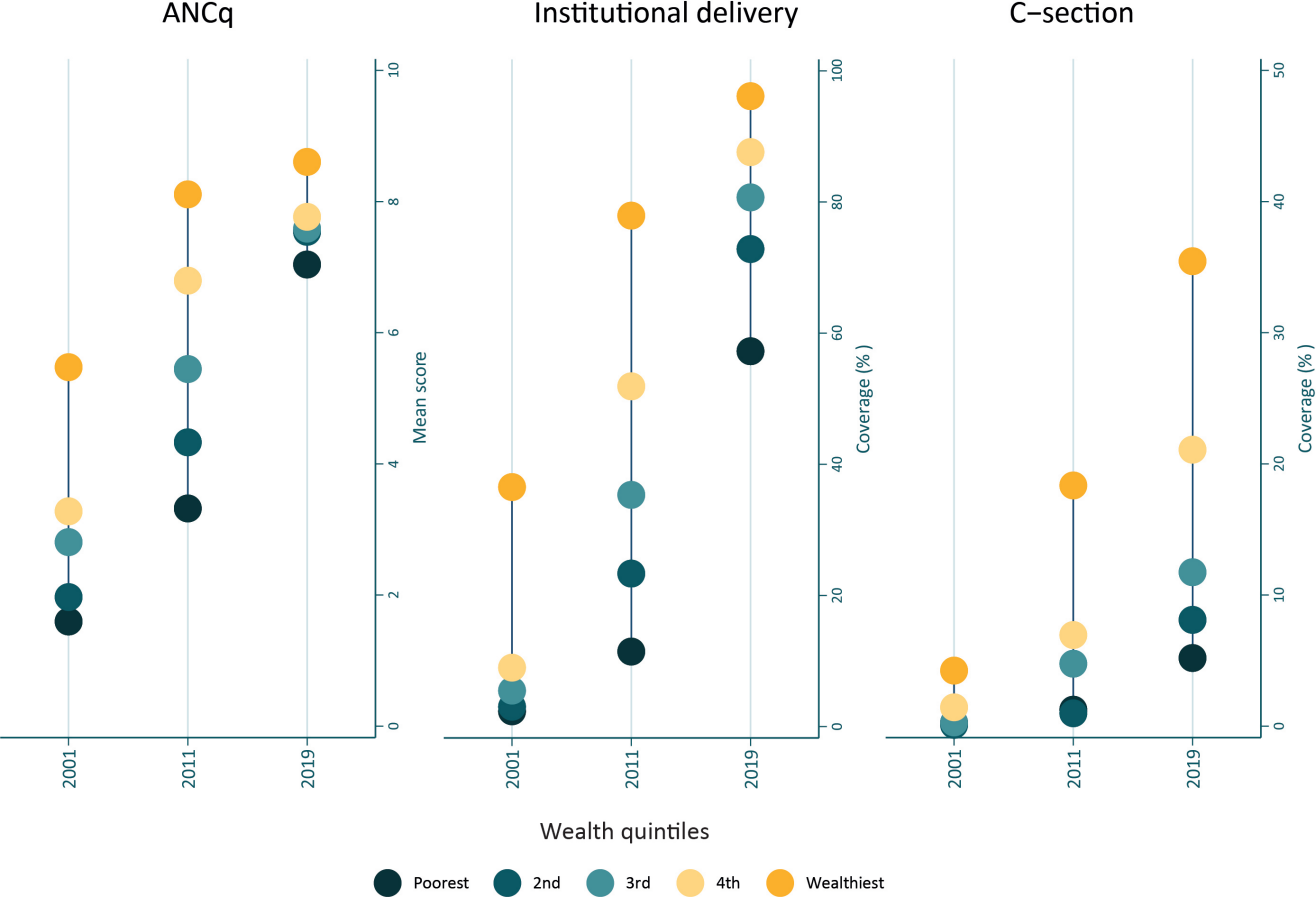
Equiplots showing use of ANCq, institutional delivery and caesarean section by wealth quintile using DHS and MICS. ANCq is a survey-based ANC indicator calculated as a score, composed of seven variables and is intended to incorporate measures of utilisation and quality. ANC, antenatal care; DHS, Demographic and Health Surveys; MCIS, Multiple-Indicator Cluster Surveys.

Figure 3 shows that coverage of ANCq, institutional delivery and caesarean sections increased for all populations, but inequalities still remained. ANCq, a measure that attempts to look at both quality and utilisation,^13^ shows clearly that access and quality of services have increased for all. Between 2001 and 2019, the inequality gap also narrowed significantly for institutional delivery, largely driven by increased access by poorer women. In 2001, it was only the wealthiest who delivered in facilities in significant numbers, but by 2019, nearly 60% of the poorest women were delivering in facilities. Although the gap between the richest and poorest narrowed significantly—but there remains the challenge that richer women were more likely to access (better quality) hospital services, while poorer women accessed lower level facilities.

The caesarean-section picture shows that although access improved for the poorest—it was still quite low, with the poorest and second poorest quintile having a caesarean-section rate lower than the 5%–15% that is often mentioned as a way of measuring access. Caesarean-section rates in the wealthiest quintile were closer to 40%, which suggests a problem of too many caesarean sections and may reflect the growth of the private sector in Nepal in the last decade. Future predictions are beyond the scope of this paper, but the unregulated private sector leading to rising caesarean section rates was a locus of concern for many policymakers.

**Figure 4:**
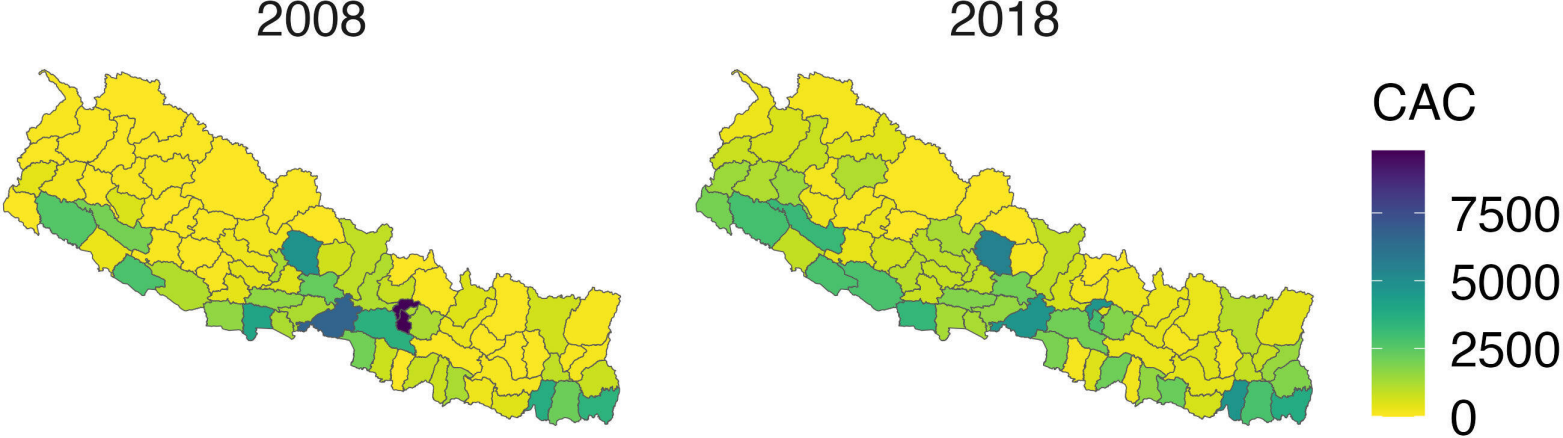
Increased access to abortion services by geographical region. CAC=comprehensive abortion care

An example of improved geographical access is seen in figure 4, which illustrates increased access to abortion services. We used HMIS to look at numbers of users of range of services, from Comprehensive Emergency Obstetric Care to abortion services and could clearly see increases across the country over the time period studied. Figure 4 shows the aspect of abortion services, and it can be seen that while services originally were only provided around the capital Kathmandu, they are now much more widely available. A similar pattern is seen with a range of other services. Despite progress, it was a common theme in interviews and focus groups that access still remained a problem for those living in very remote and rural areas.

### Mechanism 3: an approach to health policymaking and implementation that was inclusive, reflective, evidence informed, data driven and adaptive

‘We worked well together. Maybe because it was a small country. Maybe because Nepal was never colonised. Maybe because of the key individuals involved. …. We disagreed but we were friends…. We all really wanted to improve things’. *Interview with INGO Official*

Key informant interviews, focus group discussions, inception and validation meetings as well as reviews of annual reports, policy documents and literature in Nepal indicated an impressive adaptive learning and implementation process in Nepal. Nepal generally followed global evidence and trends in terms of MNH interventions. However, in reviewing the policy and implementation process over the 20-year time period, it was possible to see that there was a process of constant reflection on data, use of pilot programmes, concern about implementation, commissioning of both local and international research and action taken based on research results. There was a process of collecting information, deliberating on this information and then acting on findings and conclusions. This process is illustrated in table 1, which documents the process of developing a programme of financial incentives for women in Nepal—one that evolved over time in response to DHS/MICS results, programme evaluations, results of pilot studies and changing conditions. The policy process was not one where there were key policies adopted at certain times—but rather a constant adaption to shared goals of providing good quality care for all. There was clear evidence of this process happening in a range of policy areas.

**Table 1:** Safe Delivery Incentive Programme (SDIP) and its evolution as the Aama Programme

Year	Name	Component	Research/data identified as informing the decision
2005	Maternity Incentive Scheme	Women receive transport incentive to deliver in health facilities. Health workers receive incentives to attend deliveries at home and in health facilities.	2001 DHS showed only 10% of women delivered at facilities.^26^
2006	Safe Delivery Incentive Programme	Free delivery in 25 districts with low human development index (HDI). Health facilities reimbursed Rs 1000 per normal and complicated delivery.	Although utilisation improved overall, poorer women in low-HDI districts found service costs prohibitive.^27 28^
2009	Aama Surkshya Programme (Aama Programme)	National roll-out of free delivery care. User fees were removed from all deliveries including caesarean-sections.	Reimbursement to health facilities varied by level of care and whether the delivery was normal, complicated or with cesarean section. Early experiences documented.^28 29^
2012	Aama Programme	Separate demand-side financing added to promote four ANC visits	DHS showed ANC four had continued to be low despite service expansion.^30^
2016	Aama Newborn	Separate demand-side financing added for free newborn care.	To improve newborn care as per Nepal’s Every Newborn Action Plan.^31^
2017	Aama Programme	Free sick newborn care was removed and run seperately.	Study suggested revision based on appropriate reimbursement and extra staff for services. ^15^;^32^
2018	Aama Programme	Transport support costs were doubled across the country.	Considering higher cost of transportation prevailing at that time.

ANC, antenatal care; DHS, Demographic and Health Surveys.

Abortion policy followed a similar pattern. Abortion was legalised in 2002, and initially, starting in 2004, only doctors were trained, but services were soon expanded by training nurses. With the advent of medical abortions, assistant nurse midwives were trained to provide medical abortion. At the start of the programme, fees were charged but then dropped after concerns about affordability. Necessary equipment and drugs were added to the essential drug list over time and services were allocated budget lines. In both national and district interviews and focus groups, health workers reflected on how they saw deaths and morbidity due to illegal-induced abortions at the beginning of their careers and that now medical abortion was widely available, such cases were not seen. Nurses in the districts reflected that they were happy that they could now provide abortion services.^19^,^21^ At all stages, relevant questions on induced abortion were introduced into surveys as well as HMIS data sets to allow the government to monitor progress.

The MoHP has a mandatory period of annual review and reflection—resulting in the production of the annual report, or key policy/ strategy briefs—where key players, led by the Nepali Government (at national, regional and district level), and involving professional organisations, researchers, UN agencies, donors and international and national NGOs come together, reflect on the latest data, on what has worked and what has not, review new global evidence and strategise on how best to improve MNH. An analysis of the annual reports and government documents clearly illustrate the reflections on the latest global evidence, HMIS data, population surveys and national and international research. The Nepal Health Research Council works closely with the MoHP in commissioning research, ensuring research reaches policy makers and strengthening the research community in Nepal.

Another key factor to emerge from interviews and document reviews was the role of a range of institutions and players alongside the MoHP in developing policy. Nepal’s sector-wide approach was reported as working well, with the annual reports illustrating collaboration and involvement of many partners. Interviews with Nepali government officials and former international agency staff suggested that long-term good relationships were important in terms of progress. Some interviewees suggested that because Nepal had never been colonised, this facilitated constructive relationships. Many key Nepali players studied public health and spent some time working abroad and stated that they had learnt from experiences in countries such as India (legalisation of abortion), China, the UK and the USA. Professional organisations such as the Nepal Society of Obstetrics and Gynaecology, the Nursing Council and the Nepali Medical Council were also identified as key players that played a key role in improving MNH services, including by showing flexibility and taking a development approach to roles of different health cadres and task shifting, rather than taking protectionist attitudes and solely defending their professional interests, as seen in some other countries.

### Still more to be done

In all the interviews, focus groups and discussions while acknowledging Nepal’s achievements, the main focus and interest of those participating were on how much more needed to be achieved to provide good-quality MNH for all, including respectful care and how to achieve it. This is also reflected in planning strategies: Nepal’s Safe Motherhood and Newborn Health Road Map^15^ identified challenges that included narrowing the equity gap of women who access delivery services, ensuring that poor women were able to access caesarean sections and preventing wealthier women from having too many unnecessary caesarean sections in the growing private sector. There is also work to be done in improving services for small and sick newborns. The challenge of how to provide quality accessible services in areas with sparse populations and challenging geographies also remains and is faced in the more remote regions across the globe.

The Nepal Health Facility Survey 2021 was published after the study was completed, but found that while service availability and readiness had slightly improved from 2015, there are still huge quality gaps. The latest MMR estimates published by WHO in 2023 suggest that Nepal’s MMR now stands at 174,^22^ which is a further decrease but still suggests that Nepal has more to do.

A number of potential challenges in terms of continued success were also raised in the interviews and discussions but were outside of the scope of this study as they were the results of changes that happened in 2015 or later. The federalisation of Nepal was raised as a potential challenge to further improvements in MHN with concerns raised about its implementation for ensuring ‘that health programmes and interventions address key drivers of mortality, morbidity other areas of concern across the health systems in a concerted and coordinated manner’.^23^ While funding for MNH and health in general was historically ringfenced at a national level—this is no longer the case, and concern was expressed that municipalities may not all prioritise health. Some raised concerns about the great level of expertise that had been concentrated at regional and district level and whether this expertise would be lost. Finally, the impact of COVID on utilisation of services and on maternal and newborn outcomes, was also raised as a concern in a number of interviews.

## Conclusion

Sheikh *et al*^24^ argue that ‘learning is crucial for the performance of health systems and the achievement of broader health goals’ and that learning is more than information collecting and transfer. Action and deliberation are important—as is acknowledging what has gone wrong and correcting it. Although the language of a ‘Learning Health System’^24^ was not used by informants or in policy documents, the clear relationship between information, deliberation and action that emerged again and again when looking at policy implementation suggests that this is an appropriate description of the policy and implementation process in Nepal.

The policies and programmes that Nepal implemented to improve its MHN outcomes were not uniquely different from those of other countries that have improved maternal and newborn outcomes. What may explain how Nepal has moved rapidly from stage 2 to stage 3 in the mortality transition framework^2^ and may provide useful insights into other countries trying to do something similar is not what they did, but how they did it, with a learning approach to policy and programme development and health system strengthening.

The increase in utilisation of services, mainly driven by increasing numbers of women delivering in hospitals, talks to the global debate on how MHN services should be designed, and if the aim should be for all women to deliver in facilities which are able to provide the full emergency obstetric and newborn care.^25^

Nepal still has many of the difficulties commonly faced by countries with similar MHN indicators. Challenges remain in ensuring access for the hard to reach—whether geographical, economic or socio-cultural—and improving quality of care across the continuum of ANC, delivery, post-partum, and newborn care in an equitable and evidence-informed manner.

Finally, the approach of looking at mechanisms that have led to change over a long (20-year) period and at success brings unique methodological challenges, but also provides insights and learnings that may be missed when looking at shorter term impacts or for evidence of failures and limitations of government actions.

## Data Availability

Data used in this study include secondary data analysis of existing and publically available data sets. There were also key informant interviews and focus groups. When we requested consent for participation we stated that transcripts would not be shared beyond the research team.
